# Nummular Dermatitis Following an Insect Bite in a Patient With Activated Phosphoinositide-3-Kinase Delta Syndrome (APDS): A Case Report and Literature Review

**DOI:** 10.7759/cureus.112976

**Published:** 2026-07-19

**Authors:** Adriana Hernandez-Carrasco, Irving Fuentes-Calvo, Sara Fernanda Arechavala-Lopez, Valeria Hernandez-Carrasco, Yaara Lucila Soriano-Hernandez, Arturo García-Galicia, Zaira Aketzali Contreras-Sánchez, Gabriela Carrasco-Trinidad

**Affiliations:** 1 Neurology, Instituto Nacional de Neurología y Neurocirugía, Mexico City, MEX; 2 Surgery, Hospital Médica Sur, Mexico City, MEX; 3 Anesthesiology, Hospital Médica Sur, Mexico City, MEX; 4 Medicine, Universidad Popular Autónoma del Estado de Puebla, Puebla de Zaragoza, MEX; 5 Dermatology, Centro Médico Nacional "Gral. de Div. Manuel Ávila Camacho", Instituto Mexicano del Seguro Social, Puebla de Zaragoza, MEX; 6 Health Research, Centro Médico Nacional "Gral. de Div. Manuel Ávila Camacho", Instituto Mexicano del Seguro Social, Puebla de Zaragoza, MEX; 7 Allergy and Clinical Immunology, Centro Médico Nacional "Gral. de Div. Manuel Ávila Camacho", Instituto Mexicano del Seguro Social, Puebla de Zaragoza, MEX

**Keywords:** dermatitis, eczema, immunodeficiency, insect bites and stings, nummular, pi3k-akt-mtor signaling pathway, primary

## Abstract

Activated phosphoinositide-3-kinase delta syndrome (APDS) is a rare primary immunodeficiency caused by gain-of-function mutations in the PIK3CD gene, resulting in the hyperactivation of the PI3K-Akt-mTOR pathway and subsequent immune dysregulation. Although recurrent sinopulmonary infections are typical, cutaneous manifestations remain poorly characterized. Nummular dermatitis is an uncommon presentation in this context and may reflect the underlying inflammatory imbalance.

A 17-year-old female patient with genetically confirmed APDS (heterozygous PIK3CD c.3061G>A; p.Glu1021Lys) presented with erythematous papules that evolved into tense bullae on the lower limbs following an insect bite. Over subsequent weeks, multiple well-defined, coin-shaped eczematous plaques developed on the extremities and trunk, consistent with nummular dermatitis. The patient had a history of recurrent respiratory infections, chronic sinusitis, and otitis media, managed with intravenous immunoglobulin and prophylactic antibiotics. Topical betamethasone with fusidic acid and oral antihistamines produced partial improvement, though intermittent relapses occurred during follow-up. No systemic symptoms or new infectious complications were identified.

This case represents an unusual dermatologic manifestation of APDS, where a minor external trigger led to persistent eczema-like lesions. Dysregulated PI3K-Akt-mTOR signaling may amplify local inflammation and impair epidermal barrier recovery. Recognition of atypical or chronic dermatitis in immunodeficient patients is crucial for early diagnosis, targeted treatment, and multidisciplinary management. Reporting such cases expands the clinical spectrum of APDS and underscores the importance of considering immune dysregulation in persistent cutaneous disorders.

## Introduction

Activated phosphoinositide-3-kinase delta syndrome (APDS) is a congenital disorder characterized by the dysregulation of the immune system. PI3K is a key signaling pathway involved in immune regulation, and its alterations can result in both deficiency and hyperactivation, ultimately leading to an underlying immunodeficiency [1].

APDS is biochemically defined by the overactivation of the PI3K-Akt-mTOR signaling pathway. In APDS1, activating mutations of the PIK3CD gene, which encodes the p110δ catalytic subunit of PI3K-δ, cause a constitutive increase in PI3K-δ activity due to the alteration of inhibitory interactions with the regulatory subunit p85α [2,3]. PI3K is critical for multiple biological processes, including cell growth, differentiation, survival, proliferation, migration, and metabolism [4]. Immune dysregulation in APDS is characterized by an overabundance of immature B cells, the accumulation of senescent T cells, and a deficiency of functional immune cells [5].

Bacterial infections are highly prevalent in APDS, with approximately 80% of affected individuals developing bacterial pneumonia. Similar to other primary immunodeficiencies, an altered humoral immune response increases susceptibility to infections caused by encapsulated bacteria, which rely on antibody-mediated opsonization for effective elimination [1-6].

Although recurrent sinopulmonary infections are the hallmark of APDS, cutaneous manifestations have increasingly been recognized as part of its expanding clinical spectrum. Reported dermatologic findings include eczema, psoriasis-like dermatitis, vasculitis, recurrent viral skin infections, and cutaneous lymphoid proliferations. Nevertheless, inflammatory dermatoses remain poorly characterized in APDS. To the best of our knowledge, following a review of the available literature, this represents the first reported case of insect bite-associated nummular dermatitis in a patient with genetically confirmed APDS, highlighting a potentially underrecognized manifestation of immune dysregulation.

Nummular dermatitis, also known as discoid eczema, is a chronic inflammatory dermatosis with a recurrent course, characterized by well-defined, rounded, or oval erythematous plaques, which may present exudation, crusting, and, subsequently, xerosis or post-inflammatory hyperpigmentation. It usually follows a course of relapses and remittances and is usually associated with cutaneous xerosis, skin trauma, or hypersensitivity reactions [6]. Its occurrence in adolescents with primary immunodeficiencies is exceptionally uncommon. This case highlights a potentially underrecognized inflammatory cutaneous manifestation of APDS and expands the spectrum of dermatologic presentations associated with this rare primary immunodeficiency.

## Case presentation

The patient's clinical records and previous evaluations were thoroughly reviewed by the Immunology and Dermatology Services of Centro Médico Nacional "Gral. de Div. Manuel Ávila Camacho", Instituto Mexicano del Seguro Social, in Puebla de Zaragoza, Mexico. Clinical data, dermatological findings, and photographic documentation were collected directly during the diagnostic process and follow-up. All patient information and clinical images are published with written informed consent obtained from the patient and her legal guardian. This case report was prepared in accordance with the CARE 2017 guidelines [7].

A 17-year-old female patient, born to consanguineous parents, was the fourth child of an uncomplicated pregnancy. She had normal growth parameters and achieved appropriate psychomotor developmental milestones. Her academic performance had been satisfactory, with no evidence of neurocognitive delay.

As a pathological history, she presented with community-acquired pneumonia that required hospitalization at nine months of age, followed by recurrent respiratory infections from the age of four years. At eight years of age, she developed a persistent cough and purulent rhinorrhea, and at 12 years of age, she developed perforated otitis media, requiring tympanostomy tubes. At age 16, she underwent tonsillectomy and adenoidectomy due to right tonsillar hypertrophy, chronic pansinusitis, and recurrent oral candidiasis.

A comprehensive diagnostic approach was performed with the aim of identifying the underlying etiology of recurrent infections. Laboratory and imaging studies ruled out cystic fibrosis, immobile cilia syndrome, and autoimmune diseases. Genetic analysis identified a heterozygous pathogenic variant in the PIK3CD gene (c.3061G>A (p.Glu1021Lys)), confirming the diagnosis of APDS. Treatment with monthly intravenous immunoglobulin (IVIG) and prophylactic antibiotics was initiated, with a favorable clinical response and better control of infectious episodes.

**Figure 1:**
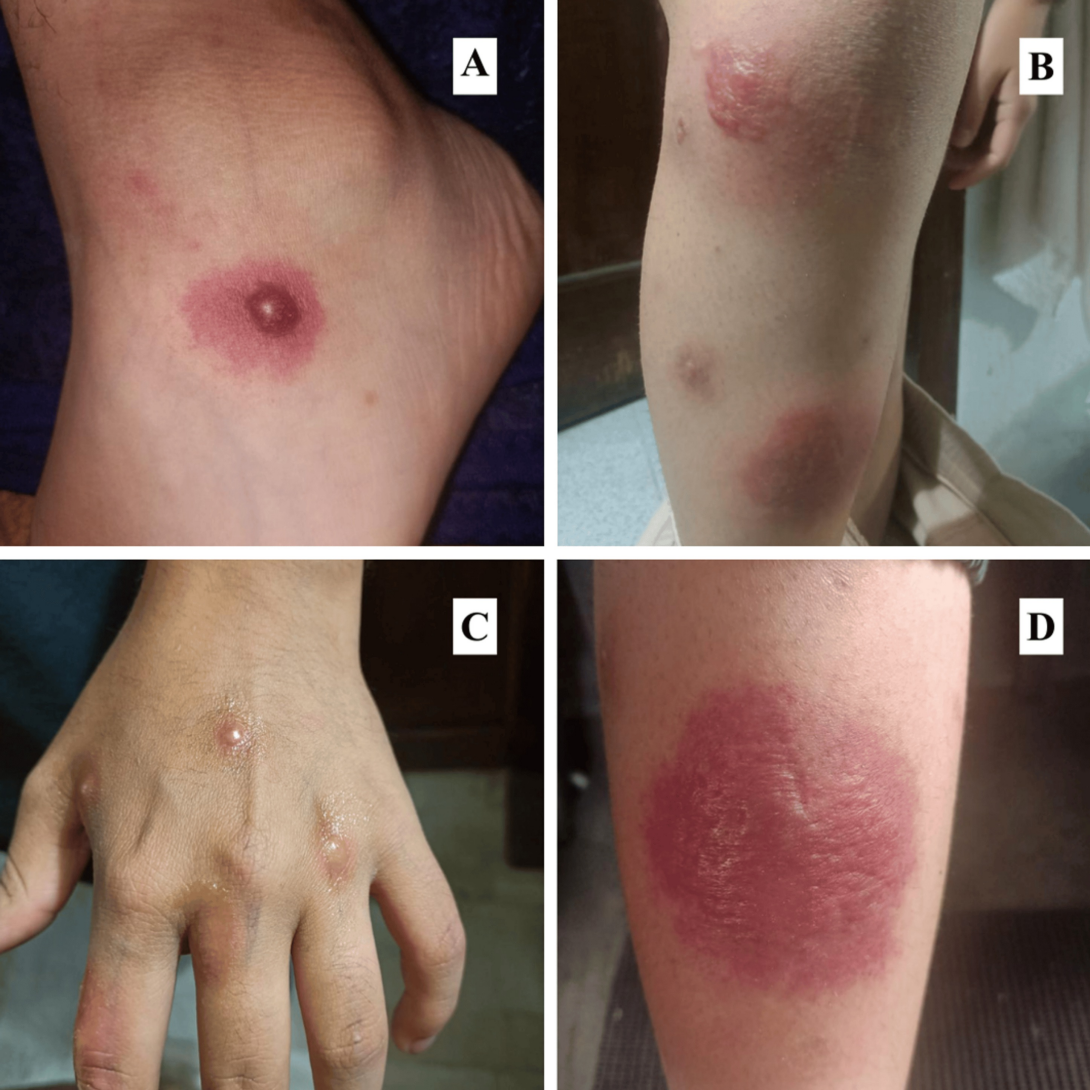
Clinical evolution of skin lesions (A) Early erythematous papular lesion with central vesiculation, located on the medial aspect of the left ankle, with a well-defined circular border and mild peripheral erythema. (B) Multiple erythematous nodular lesions with central induration on the anterior surface of the right leg, with a discrete annular configuration and initial signs of confluence. (C) Small tense blisters with serous content on an erythematous base on the dorsum of the right hand, corresponding to the acute phase of the rash. (D) Late-stage plaque on the right leg, characterized by a well-defined erythematous area, with nummular morphology, with central desquamation and mild lichenification, compatible with nummular dermatitis.

In January 2024, the patient developed an itchy erythematous papule in the left lower extremity, which progressively evolved into a taut blister with serous exudate and fine scaling, without a bad odor (Figure 1A). The initial lesion was clinically compatible with an insect bite due to its abrupt onset, the presence of a serous blister with a central punctuate area suggestive of arthropod aggression, and the patient's reported history of a sudden localized burning sensation prior to the formation of the blister. In the following days, multiple bullous lesions appeared on the right hand on an erythematous base, each with an approximate diameter of 3 mm (Figure 1C). The patient denied systemic symptoms such as fever or malaise.

At the follow-up evaluation at four months, physical examination revealed active erythematous plaques with central papules, well-defined coin-shaped borders, and signs of excoriation on the shoulders, elbows, and legs (Figure 1B-1D). No mucosal involvement or systemic manifestations were identified.

The patient was treated with topical betamethasone and fusidic acid every 12 hours for one month, oral epinastine (10 mg once daily), and vegetate-mineral thermal water compresses applied three times daily. This therapeutic scheme produced a partial clinical improvement; however, during follow-up, the patient presented intermittent relapses and remissions.

It is currently being monitored jointly by the Immunology and Dermatology Services to assess disease activity and detect possible recurrences. So far, no additional infectious or autoimmune complications have been documented.

## Discussion

This case illustrates a rare dermatologic presentation in a patient with APDS, characterized by chronic eczematous lesions that developed following an insect bite. Although APDS is mainly associated with recurrent infections and lymphoproliferative disease, skin involvement has been poorly described.

The persistence and distribution of the lesions, together with their eczematous morphology, suggest that impaired immune regulation may prolong local inflammation after a minor external stimulus. This observation supports the hypothesis that, in patients with APDS, even minimal antigenic exposure can trigger sustained inflammatory responses due to the underlying imbalance of the PI3K-Akt-mTOR pathway.

Recognition of these dermatologic changes can help clinicians identify early signs of immune dysregulation in patients with known or suspected primary immunodeficiencies. This report contributes to the growing understanding of APDS by describing a possible association between molecular activation of the immune system and chronic skin inflammation.

Dysregulation of the PI3K-Akt-mTOR signaling axis in APDS has a profound impact on cutaneous immunity, altering both adaptive immune function and epithelial barrier integrity, as well as promoting exaggerated local inflammatory responses.

The constitutive activation of the PI3K-δ isoform generates an imbalance in the lymphocyte subgroups, characterized by an excess of immature B lymphocytes, the accumulation of senescent T lymphocytes, and a reduction in functional effector populations. This alteration interferes with lymphocyte maturation and homeostasis, leading to defective adaptive responses and increased susceptibility to both immunodeficiency and autoimmunity. Likewise, the PI3K-Akt-mTOR pathway modulates the differentiation of subpopulations of T lymphocytes (Th1, Th2, Th17) and follicular helper T lymphocytes, which are essential for humoral immunity and the formation of germinal centers in cutaneous immune structures [8].

Beyond its role in immune regulation, PI3K-Akt-mTOR signaling is essential for keratinocyte proliferation and differentiation, as well as for the survival of epidermal stem cells. Its dysregulation compromises epidermal stratification and barrier function, facilitating the penetration of allergens and pathogens. This epithelial fragility has been linked to chronic inflammatory dermatoses such as atopic dermatitis and psoriasis [9,10]. In addition, Th2 cytokines can suppress key structural proteins such as filaggrin, further exacerbating skin barrier dysfunction and supporting persistent skin inflammation [8-10].

Hyperactivation of this pathway also induces aberrant keratinocyte proliferation, the excessive secretion of pro-inflammatory cytokines (IL-6, IL-8, VEGF), and the recruitment of immune cells into the skin, resulting in a persistently inflamed microenvironment [10,11]. This dysregulated response could explain the prolonged and exaggerated local inflammation observed in our patient after a minor antigenic stimulus such as an insect bite. Consequently, the cutaneous manifestations observed in APDS could reflect an imbalance between immune activation and epithelial repair mechanisms, predisposing to the development of chronic eczematous lesions even after trivial environmental exposures.

Distinguishing nummular dermatitis from other dermatological entities is critical, particularly in immunocompromised patients such as those with APDS, in whom clinical presentations may be atypical, prolonged, or complicated by superinfections. In this case, although the initial lesion appeared after a probable insect bite, the chronic evolution, the coin morphology, and the multifocal distribution favored the diagnosis of nummular dermatitis over other diagnostic possibilities.

Skeeter's syndrome, a hypersensitivity reaction to mosquito salivary antigens, usually manifests as localized edematous plaques or papules, often with vesicles or blisters, that appear acutely after the bite and are usually confined to the site of contact. Although itching can be severe, the lesions are usually self-limiting and resolve within a few days. In contrast, our patient developed progressive, symmetrical plaques, with central desquamation and without clear temporal resolution, which is not characteristic of Skeeter's syndrome [12].

Persistent arthropod bite reaction and exaggerated insect bite hypersensitivity, both of which may occur in immunocompromised individuals, were also considered. However, the chronic multifocal evolution, coin-shaped morphology, and favorable response to topical therapy were considered more consistent with nummular dermatitis.

Bullous impetigo, another potential differential diagnosis, presents with flaccid blisters containing clear or cloudy fluid that quickly rupture, leaving meliceric crusts, particularly on the face and trunk. It is usually associated with fever and lymphadenopathy. In this case, however, the absence of systemic symptoms, the chronic evolution of the lesions, and the lack of typical exudative scabs allowed this entity to be ruled out. However, the risk of secondary bacterial infection in patients with APDS remains high and requires close monitoring [12].

Atopic dermatitis typically affects flexural areas and manifests as poorly defined eczematous plaques, chronic pruritus, and a personal or family history of atopy. Although there is some overlap with nummular dermatitis, especially in patients with xerotic skin, the characteristic coin-shaped distribution, the absence of flexural predominance, and the lack of atopic history in our patient supported the primary diagnosis of nummular dermatitis [13,14].

In immunocompromised hosts, dermatoses may depart from their classic presentations, persist for prolonged periods, or become secondarily infected. Therefore, the recognition of subtle differences in morphology, distribution, and clinical evolution is essential to guide an accurate diagnosis and appropriate management. In this context, the multifocal, symmetrical plaques of nummular morphology observed, together with the absence of vesiculo-bullous changes or acute-onset features, provided solid clinical evidence in favor of nummular dermatitis (Table 1).

**Table 1:** Comparative analysis of the differential diagnoses considered for cutaneous presentation in a patient with activated phosphoinositide-3-kinase delta syndrome

Feature	Nummular dermatitis	Skeeter's syndrome	Bullous impetigo
Underlying mechanism	Chronic eczematous inflammation is associated with xerosis, epidermal barrier dysfunction, and a dysregulated immune response, particularly in immunocompromised hosts	Type I/IV localized hypersensitivity reaction to mosquito salivary antigens	Superficial bacterial infection caused by exfoliative toxins of *Staphylococcus aureus*, leading to intraepidermal cleavage
Start and clinical course	Gradual onset with chronic and recurrent evolution over weeks to months	Rapid onset, within hours of the sting; self-limited course from 3 to 10 days	Acute onset with rapid progression of vesicles to flaccid blisters over a period of 24-48 hours
Morphology of the lesions	Erythematous coin-shaped plaques, with vesiculation, scaling, and crusting; well-defined edges	Itchy erythematous papules or blisters surrounded by edema and localized swelling	Flappy blisters that break easily, leaving meliceric crusts
Anatomical distribution	Symmetrical; it most frequently affects the extremities and trunk	Limited to exposed areas such as the limbs and face	Predominantly perioral, perinasal, or in intertriginous regions
Systemic manifestations	Absent or limited to mild pruritus and xerosis	Occasionally low-grade fever or malaise	Possible fever, regional lymphadenopathy, or leukocytosis
Histopathological pattern	Spongiotic dermatitis with superficial perivascular lymphocytic infiltrate and occasional eosinophils	Dermal edema with mixed infiltrate of eosinophils and lymphocytes	Formation of subcorneal pustules with neutrophilic infiltrate and bacterial colonies
Therapeutic response	Gradual resolution with topical corticosteroids, emollients, and antihistamines	Rapid improvement with topical antihistamines and corticosteroids; avoiding new bites prevents recurrences	Marked improvement after systemic or topical antibiotic treatment

Only a limited number of publications have addressed skin involvement in APDS. Most describe systemic manifestations such as infections, lymphoid hyperplasia, or autoimmunity, mentioning skin findings briefly and often without detailed characterization.

A review of the available literature identified some reports of APDS with inflammatory or vasculitic lesions, including those of Crisanto-López et al., Yin et al., and Lu et al. [15-17]. However, none described a chronic eczematous pattern or lesions compatible with nummular dermatitis. In this context, our case provides a distinctive presentation, documenting a persistent eczematous-type rash after an insect bite in a patient with a confirmed variant of PIK3CD.

Comparable dermatological patterns have been described in other primary immunodeficiencies, in which impaired immune regulation contributes to sustained skin inflammation and altered repair mechanisms [16,17].

Despite the growing recognition of APDS, dermatological manifestations remain insufficiently described in both clinical reports and research studies. Most publications emphasize systemic complications, leaving the skin spectrum poorly defined. Greater dissemination of dermatological presentations, including chronic eczematous lesions, could help refine phenotypic classification and improve the identification of immune dysregulation through skin findings. Future studies should evaluate whether selective PI3K-δ inhibition with leniolisib improves inflammatory skin manifestations in APDS. Although leniolisib has demonstrated clinical benefit in controlling immune dysregulation and lymphoproliferation, its impact on cutaneous manifestations remains poorly characterized [18].

The cutaneous manifestations in APDS do not represent isolated superficial findings, but rather reflect underlying immune dysregulation and may act as early indicators of immunodeficiency, systemic inflammation, or even malignant transformation. Patients with APDS frequently present with recurrent skin infections, chronic dermatitis, or inflammatory skin diseases such as atopic dermatitis or psoriasis, all of which are associated with dysregulated PI3K-Akt-mTOR signaling [11,18]. Recognition of these lesions can provide valuable clinical clues, particularly in undiagnosed individuals, and can also alert to systemic complications such as severe infections or lymphoproliferative disease [19].

A summary of the most relevant reports is presented in Table 2.

**Table 2:** Case reports and relevant small series on APDS and dermatoses related to insect bites APDS: activated phosphoinositide-3-kinase delta syndrome; EBV: Epstein-Barr virus; HLH: hemophagocytic lymphohistiocytosis

Authors	Title	Year	Journal	Country	N	Age	Sex	Clinical phenotype (cutaneous/inflammatory)	Molecular variant reported?	Specific variant (if reported)
Crisanto-López et al. [15]	Case Report on Activated PI3K-Delta Syndrome	2024	Medical Bulletin of the Children's Hospital of Mexico	Mexico	1	17 years	Female	APDS with recurrent infections; undetailed skin findings	Yes	PIK3CD pathogenic variant (not specified)
Lu et al. [17]	Activating PIK3CD Mutation in Patients Presenting with Granulomatosis with Polyangiitis	2021	Frontiers in Immunology	China	2	4.5 and 7 years	Male/female	Systemic vasculitic phenotype; eczema is not reported	Yes	PIK3CD c.3061G>A (p.Glu1021Lys, E1021K)
Yin et al. [16]	First Occurrence of Plasmablastic Lymphoma in Activated Phosphoinositide 3-Kinase δ Syndrome	2021	Frontiers in Immunology	China	1	Not specified	Not specified	APDS-associated lymphoproliferative neoplasm	Yes	PIK3CD c.3061G>A (p.E1021K)
Martínez-Blanco et al. [20]	Nummular Dermatitis: Report of Two Cases in Children	2016	Argentine Archives of Pediatrics	Spain	2	5 and 7 years	Male/female	Classic nummular dermatitis with chronic recurrent course	No	-
Bonamonte et al. [21]	Nummular Contact Eczema: Presentation of a Pediatric Case	2019	The Open Dermatology Journal	Italy	1	8 years	Not specified	Nummular contact eczema with well-defined plaques	No	-
Pérez-Vanzzini et al. [22]	Hypersensitivity to Mosquito Bite Manifested as Skeeter Síndrome	2015	Revista Alergia México	Mexico	2	6 and 9 years	Male/female	Local reactions of exaggerated hypersensitivity; transient erythematous blisters	No	-
Meucci et al. [23]	Omalizumab for Prevention of Anaphylactic Episodes in a Patient with Severe Mosquito Allergy	2021	Clinical Case Reports	Italy	1	51 years	Male	Severe allergy to mosquito bites with recurrent phylaxis; good response to omalizumab	No	-
Dupuy et al. [24]	A Case of Severe Mosquito Bite Allergy Complicated by Fatal Hemophagocytic Lymphohistiocytosis	2022	Pediatric Dermatology	United States	1	14 years	Male	Severe hypersensitivity to mosquito bites complicated by EBV-related HLH	No	-

In this case, the patient's chronic and multifocal nummular dermatitis developed after a suspected insect bite and persisted despite the absence of systemic symptoms. The favorable response to standard topical treatment highlights the benefit of early recognition and localized management, guided by the immunological context. This underscores the importance of integrating dermatological evaluation into the longitudinal follow-up of patients with APDS.

A multidisciplinary approach, combining the expertise of immunologists, dermatologists, infectious disease specialists, and other specialists, is essential to optimize outcomes in APDS. This collaboration facilitates the precise differentiation between infectious, inflammatory, and neoplastic skin processes [19], allows for individualized therapeutic decisions that balance immune modulation with infection control, and supports the implementation of targeted therapies, such as PI3K-δ inhibitors, with adequate surveillance for potential skin adverse effects [11,19]. In our patient, coordinated follow-up between dermatology and immunology allowed for timely diagnosis, effective topical treatment, and adequate surveillance to detect recurrence or progression.

Ultimately, dermatologic findings in APDS should not be interpreted in isolation. On the contrary, they are valuable clinical markers of disease activity and immune dysfunction. Early identification and management within a multidisciplinary framework are critical not only to improve skin outcomes but also to reduce long-term complications and improve overall quality of life.

This case should be interpreted within the context of the available clinical setting. Histopathological confirmation and comprehensive immunologic characterization were not available during the patient's evaluation. Nevertheless, the diagnosis was supported by the clinical morphology, chronological evolution of the lesions, dermatologic assessment, and response to treatment. Although the temporal association between the insect bite and the onset of dermatitis is noteworthy, it should be interpreted as an association rather than evidence of causality.

## Conclusions

This case expands the recognized dermatologic spectrum of APDS by documenting chronic nummular dermatitis temporally associated with an insect bite in a patient with genetically confirmed disease. While this temporal relationship does not establish causality, it raises the possibility that minor environmental exposures may precipitate persistent inflammatory skin responses in genetically susceptible individuals with dysregulated PI3K signaling. Recognition of these atypical cutaneous manifestations may contribute to earlier diagnosis, refine the phenotypic characterization of APDS, and support timely multidisciplinary management.

## References

[1] Brodsky NN, Lucas CL (2021). Infections in activated PI3K delta syndrome (APDS). Curr Opin Immunol.

[2] Angulo I, Vadas O, Garçon F (2013). Phosphoinositide 3-kinase δ gene mutation predisposes to respiratory infection and airway damage. Science.

[3] Lucas CL, Kuehn HS, Zhao F (2014). Dominant-activating germline mutations in the gene encoding the PI(3)K catalytic subunit p110δ result in T cell senescence and human immunodeficiency. Nat Immunol.

[4] Okkenhaug K, Vanhaesebroeck B (2003). PI3K in lymphocyte development, differentiation and activation. Nat Rev Immunol.

[5] Lucas CL, Zhang Y, Venida A (2014). Heterozygous splice mutation in PIK3R1 causes human immunodeficiency with lymphoproliferation due to dominant activation of PI3K. J Exp Med.

[6] Hagenström K, Müller K, Klinger T, Willers C, Eyerich K, Augustin M (2026). Epidemiology of nummular eczema - methodological approaches and outcomes from nationwide claims data analyses. J Dtsch Dermatol Ges.

[7] Riley DS, Barber MS, Kienle GS (2017). CARE guidelines for case reports: explanation and elaboration document. J Clin Epidemiol.

[8] Preite S, Gomez-Rodriguez J, Cannons JL, Schwartzberg PL (2019). T and B-cell signaling in activated PI3K delta syndrome: from immunodeficiency to autoimmunity. Immunol Rev.

[9] Mercurio L, Albanesi C, Madonna S (2021). Recent updates on the involvement of PI3K/AKT/mTOR molecular cascade in the pathogenesis of hyperproliferative skin disorders. Front Med (Lausanne).

[10] Teng Y, Fan Y, Ma J (2021). The PI3K/Akt pathway: emerging roles in skin homeostasis and a group of non-malignant skin disorders. Cells.

[11] Roy T, Boateng ST, Uddin MB (2023). The PI3K-Akt-mTOR and associated signaling pathways as molecular drivers of immune-mediated inflammatory skin diseases: update on therapeutic strategy using natural and synthetic compounds. Cells.

[12] Leung AK, Lam JM, Leong KF, Leung AA, Wong AH, Hon KL (2020). Nummular eczema: an updated review. Recent Pat Inflamm Allergy Drug Discov.

[13] Hüppop F, Dähnhardt-Pfeiffer S, Fölster-Holst R (2022). Characterization of classical flexural and nummular forms of atopic dermatitis in childhood with regard to anamnestic, clinical and epidermal barrier aspects. Acta Derm Venereol.

[14] Kasap N, Kara A, Celik V (2023). Atypical localization of eczema discriminates DOCK8 or STAT3 deficiencies from atopic dermatitis. J Clin Immunol.

[15] Crisanto-López IE, Pérez-Arzola AA, Hernández-Castañeda Y (2024). Case report on activated PI3K-delta syndrome. Bol Med Hosp Infant Mex.

[16] Yin Z, Tian X, Zou R, He X, Chen K, Zhu C (2021). First occurrence of plasmablastic lymphoma in activated phosphoinositide 3-kinase δ syndrome. Front Immunol.

[17] Lu M, Gu W, Sheng Y, Wang J, Xu X (2021). Activating PIK3CD mutation in patients presenting with granulomatosis with polyangiitis. Front Immunol.

[18] Wang Y, Ma Z, An Z, Zhang Y, Feng X, Yu X (2023). Risk of cutaneous adverse events in cancer patients treated with phosphatidylinositol-3-kinase inhibitors: a systematic review and meta-analysis of randomized controlled trials. Cancer Med.

[19] Conti F, Moratti M, Sabattini E, Zinzani PL (2024). Expert insights on Hodgkin's lymphoma development in an activated PI3K delta syndrome patient undergoing leniolisib treatment. Front Immunol.

[20] Martínez-Blanco J, García-González V, González-García J, Suárez-Castañón C (2016). Nummular dermatitis: report of two cases in children [Article in Spanish]. Arch Argent Pediatr.

[21] Bonamonte D, Filoni A, Gullo G, Vestita M (2019). Nummular contact eczema: presentation of a pediatric case. Open Dermatol J.

[22] Pérez-Vanzzini R, González-Díaz SN, Arias-Cruz A (2015). Hypersensitivity to mosquito bite manifested as Skeeter síndrome [Article in Spanish]. Rev Alerg Mex.

[23] Meucci E, Radice A, Fassio F, Iorno ML, Macchia D (2021). Omalizumab for prevention of anaphylactic episodes in a patient with severe mosquito allergy. Clin Case Rep.

[24] Dupuy E, Afifi L, Jonas SJ, Cheng CE, Hogeling M (2022). A case of severe mosquito bite allergy complicated by fatal hemophagocytic lymphohistiocytosis. Pediatr Dermatol.

